# Case report: Successful treatment of a rare case of combined parathyroid adenoma, cervical bronchogenic cyst, and tracheal diverticulum with gasless endoscopic resection of neck masses *via* an axillary approach: A case report and literature review

**DOI:** 10.3389/fonc.2022.947422

**Published:** 2022-09-23

**Authors:** Dong-Ning Lu, Wan-Chen Zhang, Chuan-Ming Zheng, Ming-Hua Ge, Jia-Jie Xu

**Affiliations:** ^1^ Second Clinical Medical College, Zhejiang Chinese Medical University, Hangzhou, China; ^2^ Otolaryngology & Head and Neck Center, Cancer Center, Department of Head and Neck Surgery, Zhejiang Provincial People’s Hospital (Afliated People’s Hospital, Hangzhou Medical College, Hangzhou, China; ^3^ Key Laboratory of Endocrine Gland Diseases of Zhejiang Province, Hangzhou, China

**Keywords:** cervical bronchogenic cyst, minimally invasive surgery, axillary approach, tracheal diverticulum, parathyroid adenoma

## Abstract

Parathyroid adenoma (PA), one of the most common causes of hyperparathyroidism, generally involves a single parathyroid gland and is manifested as hyperparathyroidism. Bronchogenic cysts are rare congenital cystic lesions caused by a development malformation in bronchi during the embryonic period, which mostly occur in the lung and mediastinum, with an extremely low morbidity rate in the neck. A 27-year-old young female was found to suffer from hyperparathyroidism on routine physical examination, and further examination suggested a cystic lesion in the right inferior parathyroid area combined with a tracheal diverticulum. Therefore, she was initially diagnosed with cystic hyperplasia of the parathyroid glands complicated by a tracheal diverticulum. Gasless endoscopic resection of neck masses *via* an axillary approach was performed because of the high requirements for the surgical cosmetic effect of the patient. During the surgery, we observed that the preoperatively diagnosed cystic lesion was a combination of two masses, which were successfully resected under endoscopy. Based on the postoperative pathology and clinical features, the patient was eventually diagnosed with a rare case of triple diseases including PA, cervical bronchial cyst, and tracheal diverticulum. Now, the patient recovered well as per the follow-up with no signs of recurrence and was extremely satisfied with the cosmetic effect of the surgery.

## Introduction

PA is the most frequent cause of hyperparathyroidism, accounting for 80-85% of cases, which is usually manifested by elevations in serum parathyroid hormone (PTH) and serum calcium levels (1). Bronchogenic cysts are congenital cystic lesions of the bronchus that occur preferentially in the mediastinum and lung. Cervical bronchogenic cysts (CBCs) are unusual and have been reported predominantly in children, with only a very few cases reported in adults (2). Little is known about the typical clinical features of CBCs in adults, with over 80% of reported cases occurring in thyroid and paratracheal regions. In this context, CBCs in adults are often misdiagnosed as other neck lesions such as thyroglossal duct cysts, branchial cleft cysts, and thyroid or parathyroid tumors, which contributes to difficulties in accurate diagnosis and treatment (3). Surgery is the principal treatment option for PA and CBCs. Currently, with the accuracy of preoperative positioning modalities and the advance in surgery, an increasing number of patients and physicians prefer to use minimally invasive endoscopy for surgery. However, there is no report on the resection of CBCs through minimally invasive endoscopy. Here, we present a case of triple diseases including PA, CBCs, and tracheal diverticulum treated by gasless endoscopic resection of neck masses *via* an axillary approach.

## Case presentation

**Figure 1:**
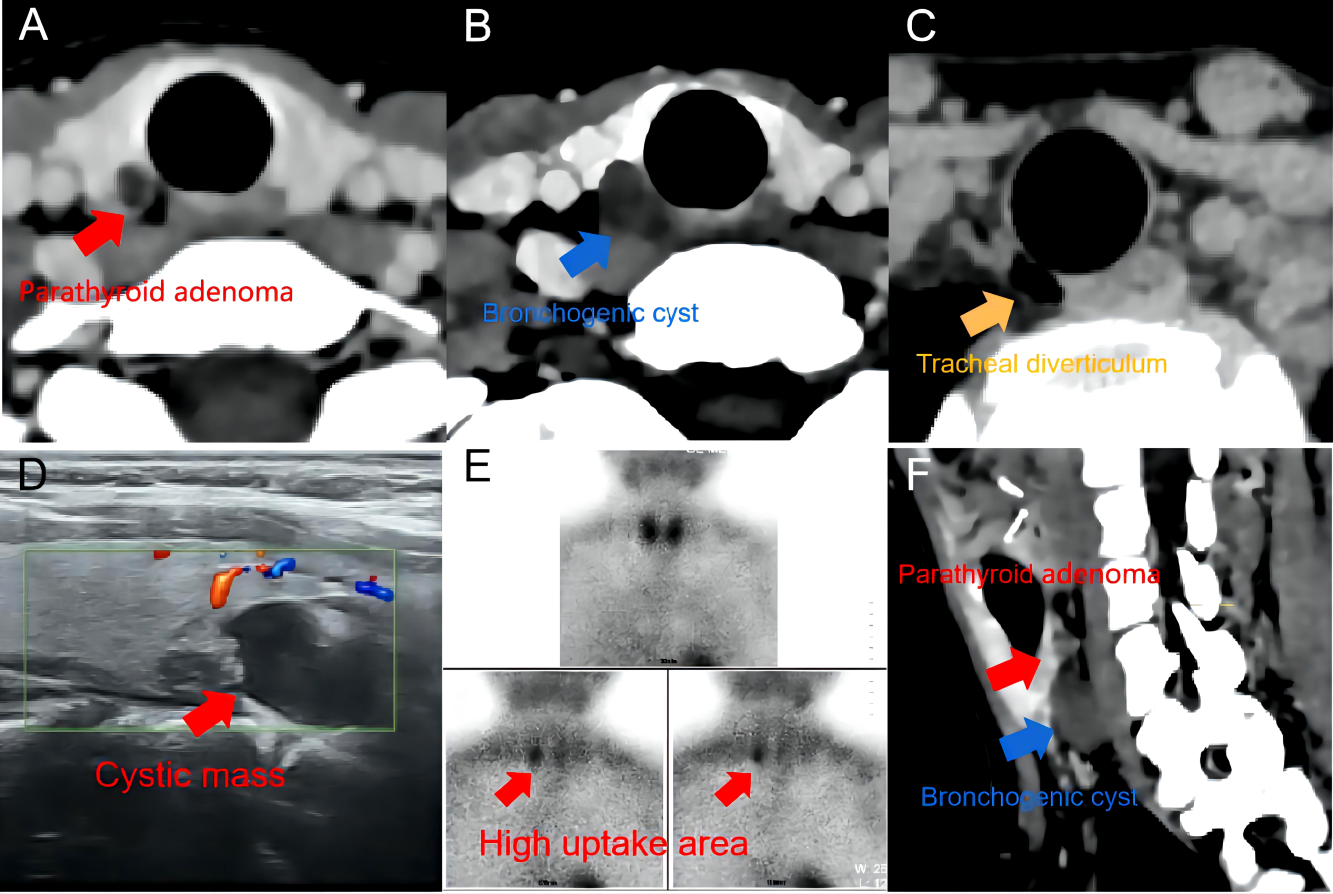
Imaging examination. **(A–C)** adenoma, cervical bronchogenic cyst, and tracheal diverticulum in CT. **(D)** a hypoechoic nodule in ultrasound. **(E)** 99mTc-sestamibi single-photon emission computed tomography: Delayed uptake (120min) in the lower panel suggests a parathyroid source. **(F)** CT multiplanar reformation (CT-MPR) of the neck.

A 27-year-old female without obvious symptoms was found to experience hyperparathyroidism on routine physical and blood examination, with a serum PTH level of 138 pg/mL and a serum calcium level of 2.56 mmol/L. Therefore, she visited our center. Ultrasound showed a hypoechoic nodule in the superficial neck with a size of approximately 11 mm * 13 mm * 15 mm visible in the right inferior parathyroid area, which was considered a disease originating from the parathyroid gland. Computed tomography (CT) of the neck indicated a tracheal diverticulum. Consequently, a benign lesion of the right inferior thyroid lobe nodule was considered, and a cyst was possible. Afterwards, ^99m^Tc-sestamibi single-photon emission computed tomography (^99m^Tc-MIBI SPECT) manifested that the delayed uptake of tracer (120min and 180min) was slightly higher in the right inferior thyroid gland (**Figures 1A–E**). Based on the aforementioned findings, the patient was tentatively diagnosed with PA complicated with a tracheal diverticulum. Because the patient was a young woman with high cosmetic requirements, “endoscopic resection of the right cervical parathyroid nodule *via* an axillary approach” was proposed after the mass was positioned.

**Figure 2:**
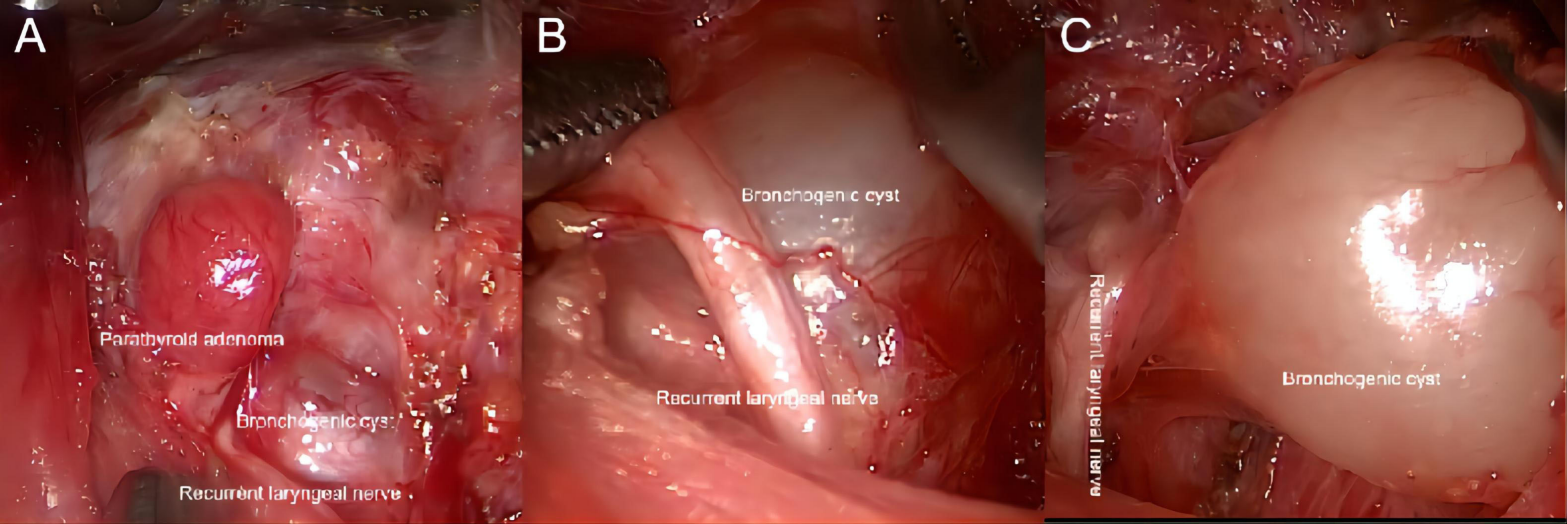
Intraoperative condition. **(A)** Intraoperative situation of PA. **(B, C)** Intraoperative situation of CBCs.

An about 4 cm-long skin incision was made in the natural fold of the right axilla and the skin was separated along the surface of the pectoralis major muscle. Then, the cavity was established by entering from the gap between the sternal and clavicular heads of the sternocleidomastoid muscle. After probing the omohyoid muscle, the anterior cervical strap muscle was further separated and pulled up to expose the right thyroid gland. It was found that there was an adenoma-like mass on the dorsal side of the inferior pole of the right thyroid gland, approximately 8 mm * 4 mm * 3 mm in size and brownish-yellow in color, with clear borders and regular morphology. A cystic mass of about 9 mm * 10 mm * 13 mm in size was found in the inferior bronchus, below the recurrent laryngeal nerve, with clear borders and regular morphology. In addition, the patient had a diverticulum in the trachea in close proximity to the cystic mass. After the informed consent was obtained from the family during the surgery, the PA and cyst were resected and the tracheal diverticulum was left untreated temporarily. Next, the middle thyroid vein was divided through coagulation, and the laryngeal recurrent nerve was located and dissected in the tracheoesophageal groove. Some tissues were collected to cover the surface of the recurrent laryngeal nerve to protect the nerve, after which the inferior parathyroid nodule was excised, followed by careful excision of the cystic nodule on the bronchus (**Figure 2**). The nerve monitor detected no diminished nerve signal in the right laryngeal recurrent nerve. PTH was detected to be 63 pg/mL during the operation and decreased by more than 50% compared with that before the operation. Hemostasis was performed, and one negative-pressure drainage tube was placed, with the resected specimen sent for examination.

**Figure 3:**
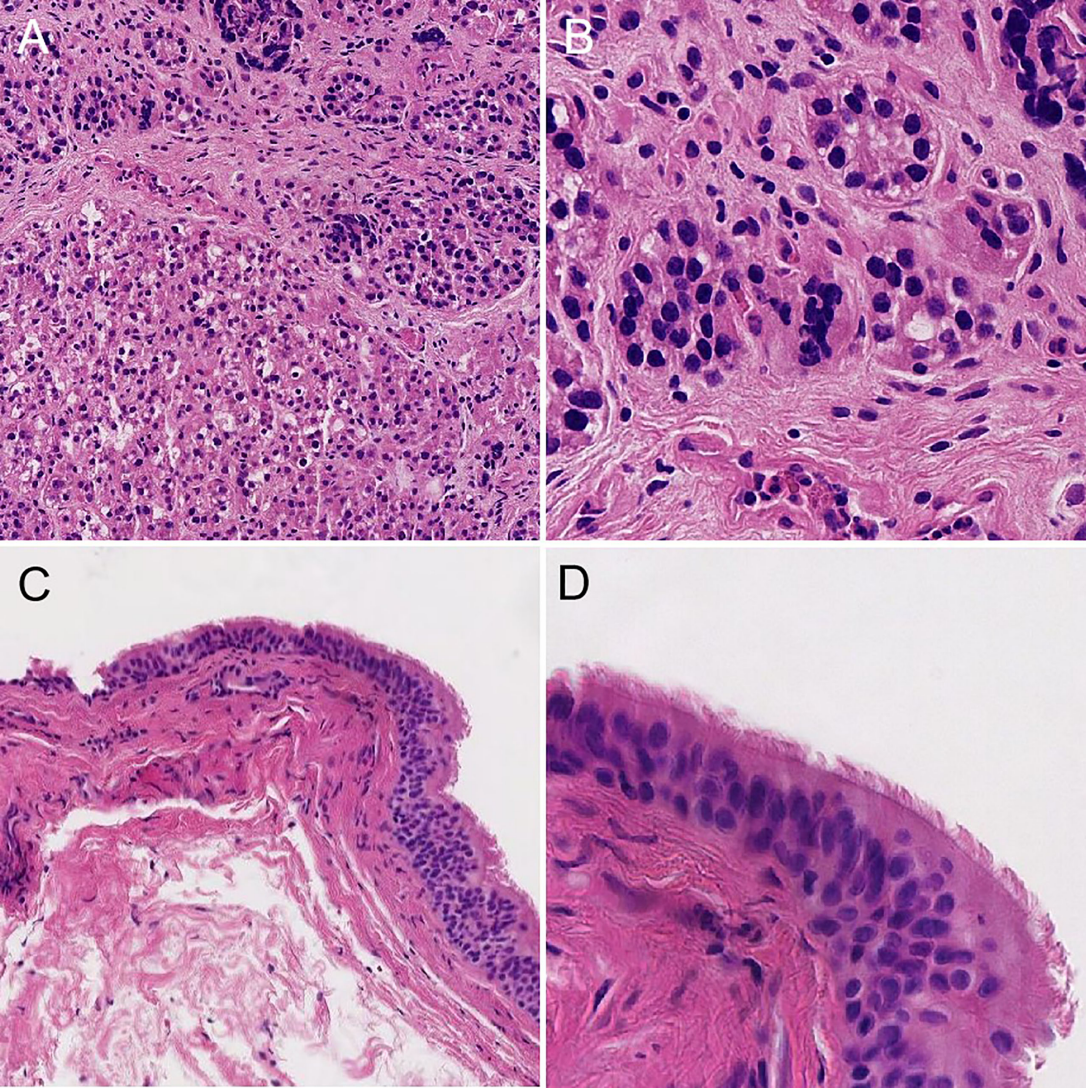
Postoperative pathology. **(A)** postoperative pathology of PA(10x). **(B)** postoperative pathology of PA(80x). **(C)** postoperative pathology of CBCs(10x). **(D)** postoperative pathology of CBCs(80x).

**Figure 4:**
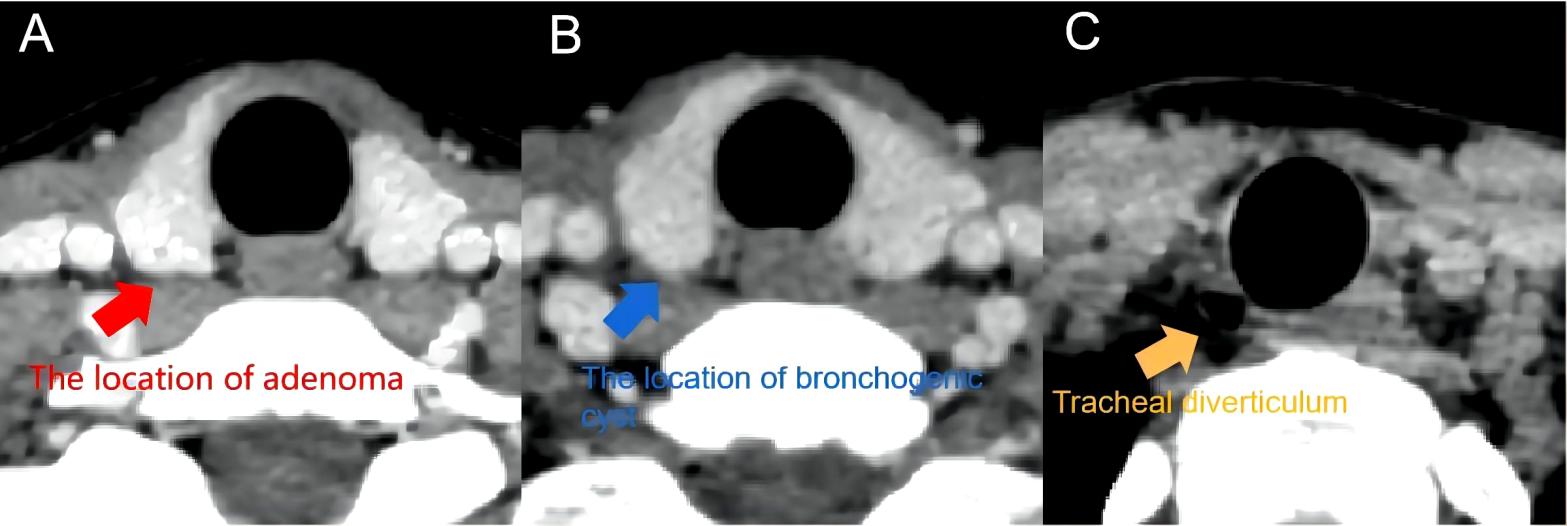
Postoperative CT. **(A)** the location of adenom in postoperative CT. **(B)** the location of cervical bronchogenic cyst in postoperative CT. **(C)** tracheal diverticulum in postoperative CT.

Postoperatively, the patient was given the usual symptomatic treatment, such as detumescence, odynolysis, and calcium supplementation, without symptoms, including fever, hoarseness, coughing and choking during drinking, and numbness of mouth and limbs. The patient was discharged on the third postoperative day after the removal of the negative-pressure drainage tube. Postoperative pathology displayed that the two masses were parathyroid adenomatous hyperplasia and a bronchogenic cyst (pseudostratified ciliated columnar epithelium is visible, **Figure 3**). Based on the clinical features of the patient combined with the surgical findings, we distinguished between parathyroid hyperplasia and adenoma and confirmed that this patient was diagnosed as a case of triple diseases of PA, bronchogenic cyst, and tracheal diverticulum. Up to now, the patient has been followed up for more than 6 months. The patient recovered well without obvious discomfort. Reexamination results showed PTH 67 pg/mL and serum calcium 3.82 mmol/L, which were all within normal levels. Neck CT indicated no recurrence in parathyroid area and no obvious change in tracheal diverticulum (**Figure 4**).

## Discussion

In the present case, hyperparathyroidism was diagnosed on routine physical examination and was empirically considered the cystic hyperplasia of parathyroid glands combined with a tracheal diverticulum based on the preoperative examination results. During the surgery, we observed a large cystic nodule in addition to an adenoma-like mass of parathyroid glands and a tracheal diverticulum, which was further identified as a rare CBC in the adult based on the postoperative pathology. Although the patient was asymptomatic, the preoperative elevation of serum calcium and serum PTH levels already suggested the progression of the disease. If the patient was not diagnosed and treated in a timely manner, symptoms such as osteoporosis and even pathological fracture might occur with long-term hyperparathyroidism. Moreover, as the bronchogenic cyst increases in size, it may cause the development of pressure symptoms and increase the risk of surgical resection at a later stage. More importantly, a prior study elucidated that CBCs also have the possibility of malignant transformation into mucoepidermoid carcinoma and malignant melanoma (4). Hence, early surgical treatment was advisable for this case.

At present, surgical resection remains the most appropriate treatment for PA, and routine preoperative localization can reduce the complications and risks of surgery (5). With the continuous progress of medical technology, increasing clinical workers try to explore ways to treat PA with less trauma. Among them, minimally invasive surgery through endoscope and ultrasound-guided thermal ablation are proved to be suitable for the treatment of specific PA (6, 7).

Bronchogenic cysts in adults, a rare cystic disease, occur in the mediastinum and lung in more than 99% of cases. Considering that minimally invasive endoscopic surgery has been widely used in clinic in recent 10 years and has made great progress, we reviewed the CBC in adults reported over the last 10 years and briefly summarized their diagnosis and treatment modalities (**Table 1**). It was observed in these reports that the majority of the patients were initially asymptomatic, with the corresponding pressure symptoms appearing as the mass was enhanced in size and a few patients experiencing co-infection. In these reports, CBCs in adults were located in almost all areas of the neck, and preoperative routine physical examination and imaging were only suggestive of cystic lesions. Because of this, almost all of the reported cases did not yield a correct diagnosis before the surgery. This is similar to the results of a large study by Jun et al. (17), which identified 18 cases with CBCs among the surgeries of 18,900 cases with thyroid cancer, all of which were misdiagnosed. Similarly, all examinations in the present case tended to suggest a cystic mass derived from parathyroid glands, and the patient was preliminarily diagnosed with cystic hyperplasia of parathyroid glands complicated with a tracheal diverticulum based on experience. Postoperatively, we looked back at all preoperative examinations and found that the bronchogenic cyst and PA could be identified only after CT multiplanar reformation (CT-MPR) of the neck (**Figure 1F**). It has been reported that although low-signal cystic masses mostly indicated by special examinations of bronchogenic cysts cannot be diagnosed definitively, the extent of the cysts and their relationship with the surrounding anatomic structures can be clarified to provide definite guidance for surgical treatment (18). For CBCs, surgical resection was utilized for tolerant patients in the prior literature. However, we found few reports of endoscopic surgery applied for CBCs in adults and only one reported case of thoracoscopic excision of thoracic bronchogenic cysts (19). After reviewing the literature, we also noted that because of the rarity and high misdiagnosis rate of CBCs, it is difficult for the attending surgeon to prepare for the surgery in advance. Additionally, the complex anatomy of the neck, multiple neurovascular, the thin wall of bronchogenic cysts, and other factors predispose to intraoperative rupture of the cyst wall, intraoperative injury to the laryngeal recurrent nerve, and other accidents, which result in complications such as infection and pus, dyspnea, and hoarseness, seriously affecting the survival quality of patients.

**Table 1:** Review of cases of CBCs in adults over the last 15 years.

First author, year	Sex	Age, year	Location	Preliminary diagnosis	Therapy	Size	Outcome
Zhengwei Gui (8), 2022	F	26	Left thyroid	Thyroid nodule	Thyroid gland lobectomy	1.0 × 1.0 cm	Reoperation, because of postoperative infection and fistula formation
	F	75	Left thyroid	Goiter	Thyroid gland lobectomy	5 × 1.5 cm	Tracheostomy, because of the dyspnea and subcutaneous emphysema of face and chest Jejunostomy because of esophageal fistula Died 125 days after the first operation
Anam Mumtaz (9), 2021	F	41	Cervical Region	Medullary thyroid carcinoma	Total thyroidectomy with neck dissection	Unspecified	Recovery well
Inês Santos (10), 2019	M	84	Infrahyoid	Thyroglossal cyst	Sistrunk	7 cm in diameter	Recovery well
Mohammed Farid (11), 2017	M	24	Midline neck	Laryngocoele	Complete excision	5 × 4 × 4 cm	Unspecified
Zhonglong Liu (12), 2016	F	70	Below the right thyroid gland	Branchial cleft cyst	Complete excision	3.3 × 3.0 cm	Recovery well
Ángel Cilleruelo Ramos (2), 2015	F	45	Below the right thyroid gland	Right-side cervical mass	Below the right thyroid gland	8 cm in diameter	Recovery well
JC Mangwiro (13), 2012	F	81	Posterior to the carina	Bronchogenic cyst	Support treatment	10.3 × 4.3 cm	Fit
Lucía Mercede Niño-Hernández (14), 2011	F	29	Hyoid midline	Thyroglossal cyst	Complete excision	2.7 × 1.4 cm	Recovery well
Alagappan Annamalai (15), 2011	M	30	Right thyroid	Thyroid nodule	Thyroid resection and exploration of the neck	5cm in dimension	Recovery well
Audrey P Calzada (16), 2011	F	32	Left thyroid	Papillary thyroid carcinoma	Total thyroidectomy and left neck dissection	4.2 × 3.5 cm	Diagnosed as a poorly differentiated adenocarcinoma arising from a cervical bronchogenic cyst, and the poorly differentiated adenocarcinoma was seen invading into the thyroid gland, skeletal muscle, and fibroadipose tissue in postoperative reexamination. The patient was taken concurrent chemoradiation with taxol and carboplatin.

Congenital tracheal diverticula are generally smaller than acquired tracheal diverticula and result from defects in endodermal differentiation during the development of the posterior tracheal wall or defects in tracheal cartilage development during the sixth week of fetal life, among which are usually not treated (20). Therefore, the tracheal diverticulum was not resected in this case. It has been noted that bronchial cysts are also a congenital disease caused by abnormal development of the trachea and bronchial tree during the embryonic period. In the current case, we speculated that there was some connection or correlation of the etiology between these two diseases due to the proximity of the location, which has not been directly validated by research. Consequently, the association of tracheal diverticula with bronchial cysts needs to be explored in more cases and studies.

**Figure 5:**
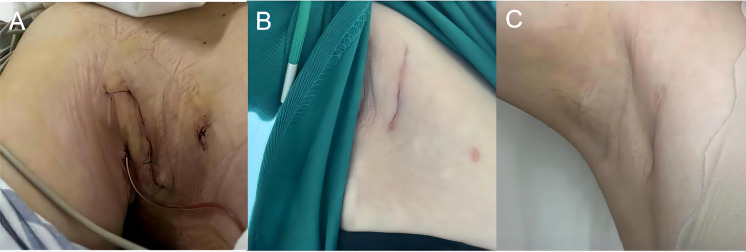
Incision condition. **(A)** Postoperative incision condition (a day). **(B)** Postoperative incision condition(a month). **(C)** Postoperative incision condition(six month).

We used gasless endoscopic resection of neck masses *via* an axillary approach for this case. Gasless endoscopic surgery *via* an axillary approach was first carried out by Chung et al. in Korea and applied for the resection of thyroid tumors (21). Gasless endoscopic surgery *via* an axillary approach was conducted in numerous medical centers because of its advantages of concealed and esthetic incision, favorable protection of the recurrent or superior laryngeal nerve under endoscopic magnification, no CO_2_-related complications, and excellent protection of the anterior cervical functional area. Likewise, the axillary approach has unique anatomic advantages over other approaches for exposing parathyroid glands. It has been documented that endoscopic surgery *via* an axillary approach has advantages of safety, rapidity, low learning cost, minimal surgical damage, and superior cosmetic results in the management of parathyroid-related diseases (22). In the present case, the visual analog scale score was 3 at the 4th hour postoperatively, and both the patient and the surgeon gave a patient and observer scar assessment scale (POSAS) score of 1. Moreover, the patient had no significant pain on review 1 month after the surgery, and the POSAS score remained at 1 (**Figure 5**). Because of the focus of this case was located in the right inferior thyroid gland, We preoperatively considered various surgical approaches and concluded that the axillary approach had the advantages of convenience, minimal damage, and superior cosmetic effects. Meanwhile, the use of this approach enabled us to precisely detect such a rare case with proximally located triple diseases under endoscopic magnification and to safely complete the resection of both masses without changing the procedure or increasing the incision. This experience suggests that gasless endoscopic resection of masses *via* an axillary approach is both operable and safe in patients with parathyroid lesions or benign cervical masses.

In summary, we reported a rare case of triple diseases including PA, CBCs in an adult, and tracheal diverticulum, which was successfully resected using a gasless endoscopic surgery *via* a unilateral axillary approach in the presence of three disease locations adjacent to each other. We can realize from this case that minimally invasive surgery plays a vital role in the treatment of head and neck tumors, and doctors can choose personalized treatment methods for individual patients to ensure the best prognosis for patients.

## Data availability statement

The original contributions presented in the study are included in the article/supplementary material. Further inquiries can be directed to the corresponding author.

## Ethics statement

The studies involving human participants were reviewed and approved by Ethics Committee of Zhejiang Provincial People’s Hospital. The patients/participants provided their written informed consent to participate in this study. Written informed consent was obtained from the individual(s) for the publication of any potentially identifiable images or data included in this article.

## Author contribution

DL was a major contributor in writing the manuscript. WZ was responsible for the acquisition of the presented data and was involved in the revision process of the manuscript. CZ was the chief surgeon and responsible for designing the manuscript. MG analyzed and interpreted the patient data. JX participated in the extensive revision process of the manuscript. All authors contributed to the article and approved the submitted version.

## Funding

National Natural Science Foundation of China (81802674).

## Conflict of interest

The authors declare that the research was conducted in the absence of any commercial or financial relationships that could be construed as a potential conflict of interest.

## Publisher’s note

All claims expressed in this article are solely those of the authors and do not necessarily represent those of their affiliated organizations, or those of the publisher, the editors and the reviewers. Any product that may be evaluated in this article, or claim that may be made by its manufacturer, is not guaranteed or endorsed by the publisher.

## Abbreviations

PA, parathyroid adenoma; PTH, Parathyroid hormone; CBC, Cervical bronchogenic cysts; CT, Computed tomography; ^99m^Tc-MIBI SPECT, ^99m^Tc-sestamibi single-photon emission computed tomography; CT-MPR, CT multiplanar reformation; POSAS, Patient and observer scar assessment scale.
